# Dissecting the polygenic architecture of psychopathology via singular value decomposition of eight psychiatric genome‐wide association studies and evaluation of component‐based polygenic scores

**DOI:** 10.1002/gps3.70027

**Published:** 2026-06-01

**Authors:** Fernando Facal, Ana M. Pérez‐Gutiérrez, Javier Costas

**Affiliations:** ^1^ Instituto de Investigación Sanitaria (IDIS) de Santiago de Compostela Complexo Hospitalario Universitario de Santiago de Compostela (CHUS) Servizo Galego de Saúde (SERGAS) Santiago de Compostela Galicia Spain; ^2^ Servizo de Psiquiatría Complexo Hospitalario Universitario de Santiago de Compostela Servizo Galego de Saúde (SERGAS) Santiago de Compostela Galicia Spain

**Keywords:** affective disorders, behavioural, biological psychiatry, factor analysis, genetics, mental disorders, psychotic, statistical

## Abstract

**Background:**

Current research suggests that genetic risk for psychiatric disorders is largely due to distinct combinations of many common variants shared by different disorders. This points to the existence of latent components affecting different dimensions of psychopathology.

**Aims:**

The aim of this study is to identify and characterise latent genetic components involved in psychopathology using a data‐driven approach and evaluate their potential as principal component‐based polygenic scores (PC‐PGSs).

**Methods:**

Singular value decomposition was applied to a matrix of summary statistics from the largest available genome‐wide association studies (GWASs) for eight psychiatric disorders to identify latent components. The components were characterised by gene mapping of the top contributing variants, enrichment analysis and genetic correlation with external traits. PC‐PGSs were evaluated in the FinnGen dataset by computing group‐wise PGSs from summary statistics using Reconstructing Allelic Count.

**Results:**

The different components were mainly involved in synapse organisation and neurodevelopment. The first latent component (PC1) explained 30.5% of the total variance and represented a broad transdiagnostic dimension. Neuroticism was the most strongly correlated external trait. Substance use traits and other psychiatric disorders were positively correlated, whereas cognitive traits were negatively correlated. The second latent component (14.7% of the variance) contrasted thought disorders with childhood‐onset neurodevelopmental disorders. Educational attainment and creativity were the most correlated external traits. Other components were more related to a single disorder or differentiated between two related disorders. PC‐PGSs in FinnGen largely showed associations in the expected direction, indicating a consistent overall trend for the different PC‐PGSs. PC1‐PGS was associated with all disorders. Other PC‐PGSs showed the expected association in case–case comparisons.

**Conclusions:**

Decomposing GWAS summary statistic matrices can reveal functionally coherent dimensions of psychiatric genetic risk that could be clinically relevant, offering a potential framework to refine diagnosis, improve prediction and inform personalised treatment.

## Abstract (highlights)

**WHAT IS ALREADY KNOWN ON THIS TOPIC**

- Psychiatric disorders share a substantial proportion of common genetic variants, suggesting the existence of latent transdiagnostic dimensions. However, previously used approaches to uncover these dimensions from genome‐wide association study (GWAS) data required an a priori specific model.

**WHAT THIS STUDY ADDS**

- This is the first study to apply singular value decomposition (SVD) to GWAS summary statistics of eight major psychiatric disorders, identifying eight coherent, interpretable latent genetic components. These components capture both shared and differential genetic dimensions and enable the generation of principal component‐based polygenic scores (PC‐PGSs) that predict psychiatric risk patterns in independent samples.

**HOW THIS STUDY MIGHT AFFECT RESEARCH, PRACTICE OR POLICY**

- Decomposing GWAS data using SVD offers a novel framework to dissect the polygenic architecture of psychopathology. This approach may enhance the biological interpretation of genetic associations and inform more personalised prediction and treatment strategies through PC‐PGSs.

## Graphical abstract

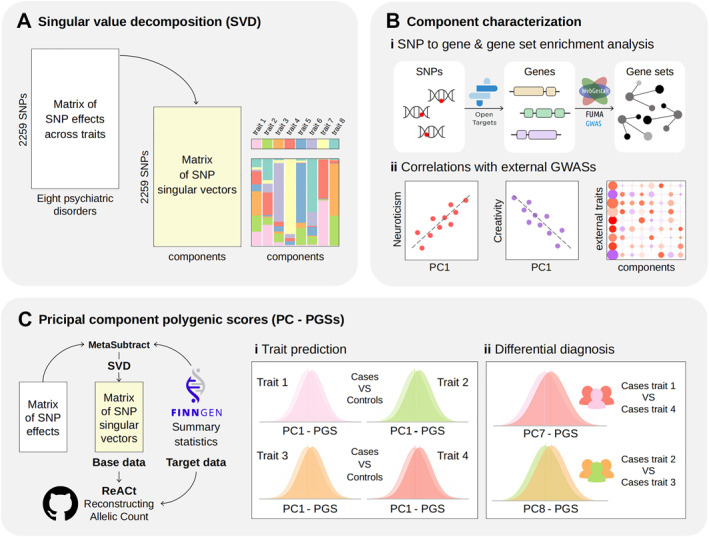

## INTRODUCTION

Genome‐wide association studies (GWASs) have revealed that psychiatric disorders are characterised by high levels of pleiotropy and polygenicity.^1^ Thousands of single nucleotide polymorphisms (SNPs) have been associated with them, many of which are shared across conditions.^2^ The shared genetic liability may, in part, reflect a general dimension of psychopathology, referred to as the *p* factor, as well as more specific latent factors encompassing groups of disorders.^3^, ^4^, ^5^, ^6^

A statistical approach increasingly applied to investigate genetic susceptibility across genetically correlated traits is genomic structural equation modelling (SEM).^7^ Genomic SEM is used to test the model fit of a priori‐defined common latent factors shared among related phenotypes. An alternative hypothesis‐free, data‐driven approach is the decomposition of GWAS summary statistics into principal components (PCs), potentially capturing both shared factors and trait‐specific factors, provided that each trait is relatively independent of the others. Specifically, singular value decomposition (SVD) has been used on matrices of SNP effect sizes derived from related GWASs to identify uncorrelated latent components (hereinafter referred to as PCs).^8^ Decomposing the phenotype‐by‐SNP effect matrix yields, for each PC, a singular value (reflecting the proportion of variance explained by the PC), a SNP singular vector (indicating the contribution of each SNP to the PC) and a phenotype singular vector (representing the contribution of each phenotype to the PC). Additionally, the contribution of each PC to individual phenotypes can be estimated. This approach enables the identification of functionally coherent groups of SNPs and the phenotypes most associated with each PC.^8^, ^9^ For instance, this approach has been used to identify PCs that account for a substantial portion of the genetic susceptibility across 159 diseases.^9^ The analysis showed that diseases classified together tend to share similar contributions from the same PCs. SVD has also been effectively used to decompose the genetic risk of specific diseases.^10^ A similar approach was applied to brain imaging GWAS summary statistics.^11^

Genomic biomarkers may play a role in precision‐guided mental health practice.^12^ Specifically, polygenic scores (PGSs), calculated as the weighted‐by‐effect sum of risk alleles, have shown promise in improving prediction accuracy in psychiatry.^13^, ^14^, ^15^ Nonetheless, their predictive utility is not yet sufficient for clinical application.^16^ One key limitation is that PGSs capture the overall genetic liability as a single score, assuming additive effects, without accounting for the diversity of underlying mechanisms, including potential interactions.^17^, ^18^ Coordinated forms of polygenic interactions may exist among different latent genetic factors,^19^, ^20^ as well as between latent genetic factors and environmental factors, as exemplified in the case of physical activity in the development of type 2 diabetes.^10^ Therefore, alternative ways to quantify polygenic risk that more accurately reflect the complexity of the underlying biological processes are warranted.^10^, ^17^, ^18^ One approach involves the calculation of PC‐PGSs, whereby SNPs are weighted by their loadings on a given PC. PC‐PGSs are derived from a purely data‐driven statistical decomposition of GWAS summary statistics and do not incorporate biological annotations during score construction. However, previous studies suggest that these PC‐PGSs have the advantage of being more biologically coherent and interpretable.^10^, ^21^

The decomposition of phenotype‐by‐SNP effect matrices thus holds considerable potential for advancing several areas. Despite its promise, this methodology has not yet been applied to psychiatric disorders, even though their pervasive pleiotropy makes them particularly suitable. The aim of this study is to apply the SVD method to the largest available GWASs of psychiatric disorders in order to identify and characterise latent genetic components and evaluate their potential as PC‐PGSs.

## METHODS

**FIGURE 1:**
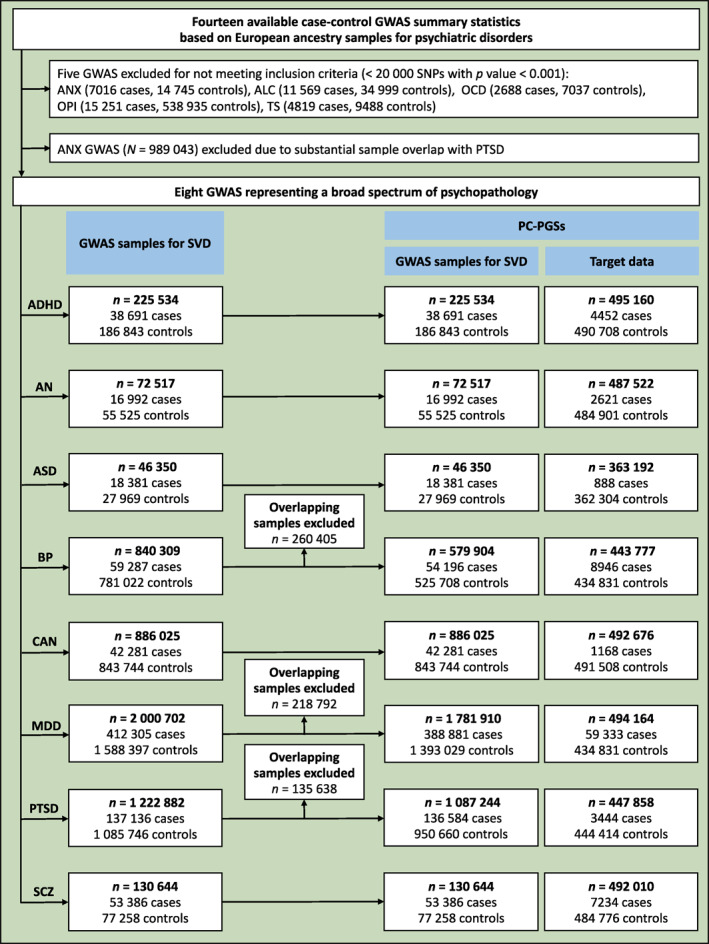
Flow chart of the data sources. The study was based exclusively on publicly available GWAS summary statistics, as detailed in table S1. GWASs included in the SVD were obtained from PGC, with the exception of CAN, which was obtained from the MVP. PC‐PGSs were subsequently evaluated using GWAS summary statistics from the FinnGen project, based on SVD of GWASs after the removal of overlapping samples. No individual‐level data were used at any stage of the analyses. ADHD, attention deficit hyperactivity disorder; ALC, alcohol dependence; AN, anorexia nervosa; ANX, anxiety; ASD, autism spectrum disorder; BP, bipolar disorder; CAN, cannabis dependence; GWAS, genome‐wide association study; MDD, major depressive disorder; MVP, Million Veteran Program; OCD, obsessive‐compulsive disorder; OPI, opioid dependence; PC‐PGSs, principal component‐based polygenic scores; PGC, Psychiatric Genomics Consortium; PTSD, post‐traumatic stress disorder; SCZ, schizophrenia; SVD, singular value decomposition; TS, Tourette syndrome.

**FIGURE 2:**
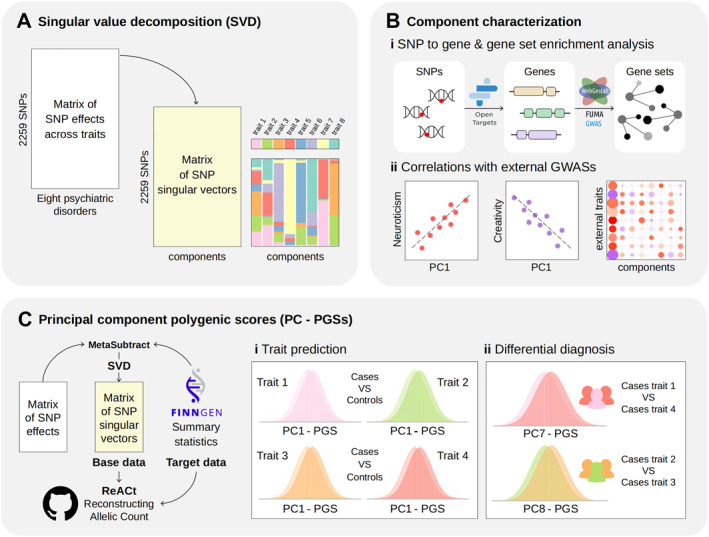
Illustrative study overview. (A) Summary statistics from GWASs of eight psychiatric disorders were harmonised and filtered to construct a SNP effect‐by‐disorder matrix. SVD was applied to extract eight orthogonal PCs. (B) The PCs were subsequently characterised by (i) performing a variant‐to‐gene mapping step (Open Targets Genetics), followed by gene set enrichment analyses of nonredundant GO biological processes (WebGestalt) and assessment of gene expression across human brain developmental stages (FUMA) and (ii) examining correlations with GWASs of external traits. (C) Polygenic scores derived from the PCs (PC‐PGSs), recalculated after extracting FinnGen data with MetaSubtract, were then computed in FinnGen samples from GWAS summary statistics using the ReACt tool to (i) evaluate trait prediction of the eight psychiatric disorders (cases vs. controls) and (ii) test hypotheses about differential diagnosis in case–case GWASs (also generated by ReACt). GO, Gene Ontology; GWASs, genome‐wide association studies; PC‐PGSs, principal component‐based polygenic scores; PCs, principal components; ReACt, Reconstructing Allelic Count; SNP, single nucleotide polymorphism; SVD, singular value decomposition.

A flow chart illustrating the selection of the analysed study sample is presented in figure 1, and an overview of the study design is shown in figure 2.

### Summary statistics and matrix construction

This study used only publicly available GWAS summary statistics; therefore, no institutional approval was needed. All original studies received ethical committee approvals. Case–control GWAS summary statistics for psychiatric disorders based on European ancestry samples were considered to ensure a comparable linkage disequilibrium (LD) structure. The Psychiatric Genomics Consortium (PGC) GWAS of anxiety,^22^ which included several anxiety‐related diagnoses such as post‐traumatic stress disorder (PTSD), was excluded due to substantial case overlap with the larger PTSD GWAS.^23^ Although the case samples across the included GWASs corresponded to distinct primary diagnoses, sample overlap cannot be entirely excluded, as individuals with comorbid conditions may have been included in more than one disorder‐specific GWAS. In addition, partial overlap of control samples across studies is well documented due to the reuse of large, shared cohorts within international consortia. In total, eight GWASs were selected, representing a broad spectrum of psychopathology: externalising disorders (attention deficit hyperactivity disorder [ADHD]^24^ and cannabis dependence [CAN])^25^; internalising disorders (major depressive disorder [MDD]^26^ and PTSD); the compulsive disorder anorexia nervosa (AN)^27^; psychotic disorders (schizophrenia [SCZ]^28^ and bipolar disorder [BP])^29^; and childhood‐onset neurodevelopmental disorders (autism spectrum disorder [ASD]^30^ and ADHD).

Filters based on statistical significance were applied during the construction of the matrix, as described in previous studies.^8^, ^9^ Following these studies, a *p* value threshold of 0.001 was applied to all GWASs to remove the most unreliable estimates of association. Those GWASs containing more than 20 000 SNPs surpassing this threshold were chosen for the analysis. This cutoff was selected based on the distribution of the number of SNPs at *p* value < 0.001 by disorder (tables S1 and S2) to maintain a balance between including a broad range of disorders and retaining an adequate number of SNPs for reliable decomposition. Genomic SEM software^7^ was used to harmonise effect alleles, using HapMap 3 as a reference panel (N SNPs = 87 270). *Z*‐scores were computed to represent effect sizes. For SNP–disorder pairs with a *p* value > 0.001, the *Z*‐score was set to zero to minimise the inclusion of unreliable associations. Only SNPs with non‐zero *Z*‐scores in at least two disorders were retained (N SNPs = 18 193). Subsequently, clumping was performed using PLINK 1.9,^31^ prioritising SNPs associated with a greater number of disorders with non‐zero *Z*‐scores (physical distance = 500 kb; *r* ^2^ = 0.2; 1000 Genomes Phase 3 European samples as a reference panel) (N SNPs = 2259). Finally, *Z*‐scores for each GWAS were standardised to have a mean of zero and a standard deviation of one. To assess the robustness of the PCs, the matrix construction was repeated in four secondary analyses. Two of them consisted of the use of different *p* value thresholds for SNP inclusion in the matrix construction (< 1 × 10^−5^ and < 0.05). In each case, the same *p* value threshold (< 1 × 10^−5^ and < 0.05) was used to set to zero the *Z*‐scores of SNP–disorder pairs not meeting that threshold. In a third secondary analysis, the major histocompatibility complex (MHC) region (chromosome 6: 25 477 797–36 448 354 base pairs, hg19) was excluded prior to clumping due to its complex pattern of LD. Finally, in a fourth secondary analysis, the three GWASs contributing most strongly to the matrix (SCZ, BP and MDD) were replaced with earlier PGC releases with smaller European sample sizes (SCZ:^32^ 33 640 cases and 43 456 controls; BP:^33^ 41 917 cases and 371 549 controls; MDD:^34^ 59 851 cases and 113 154 controls) to test the effect of different statistical powers of the selected GWASs.

### Singular value decomposition

SVD was performed using the *svd* function in *R* on a matrix of *Z*‐scores corresponding to 2259 independent SNPs across eight psychiatric disorders. The original matrix was decomposed into three components: a matrix of phenotype singular vectors, a matrix of SNP singular vectors and a diagonal matrix of singular values. The variance explained by each PC was calculated as the ratio of its squared singular value to the sum of all squared singular values. The relative contribution of each phenotype or SNP to a given PC was estimated as the squared values of the respective columns in the singular vector matrices. Finally, the relative contribution of each PC to each phenotype was calculated using squared cosine scores, as previously described.^8^

### SNP to gene annotation

Protein‐coding genes were assigned to SNPs using the Open Targets Genetics resource^35^ through the R package *otargen* ^36^, as in a previous study.^37^ For each SNP, the gene with the highest variant‐to‐gene score was selected, and all genes with transcription start sites located within 100 kb of the SNP were also considered.

### Gene set enrichment analysis

Each PC was tested for gene set enrichment using WebGestalt, implemented through the R package *WebGestaltR*.^38^ For this analysis, SNPs that cumulatively accounted for 50% of the variance of each PC were included. This threshold was selected to balance signal specificity and statistical power. As a sensitivity analysis, an alternative cumulative variance threshold of 25% was also evaluated. An over‐representation analysis of nonredundant Gene Ontology (GO) biological processes was carried out, using the ‘genome protein‐coding’ gene set as background. For over‐representation analyses, the strength of enrichment was quantified using the enrichment ratio (observed number of genes divided by the expected number under the null). The significance was tested by the hypergeometric test. The analysis was limited to GO categories containing between 20 and 500 genes. Gene sets with a false discovery rate (FDR) below 0.05 were retained for interpretation.

Gene expression across different human brain developmental stages was analysed for each PC using the BrainSpan dataset in the web‐based platform FUMA.^39^ Specifically, the GENE2FUNC tool was used to test for enrichment of differentially expressed gene sets at each of 11 general developmental stages of brain samples.

### Correlations with external GWASs

To further characterise the PCs in relation to additional traits, the SNP loadings for each PC were transformed into *Z*‐scores. Then, Pearson correlations were computed between these *Z*‐scores and the corresponding *Z*‐scores of the same SNPs from GWAS summary statistics of external traits. The external traits included psychiatric disorders that were not part of the original matrix, psychological traits, substance use phenotypes and immune‐related traits. Genomic SEM was used to harmonise effect alleles.

### PGSs in FinnGen samples

PGSs were computed using the most recent release (DF12) of the FinnGen summary statistics as the target data for the eight psychiatric disorders.^40^ Sample overlap between FinnGen and the discovery GWASs for BP, MDD and PTSD was identified (table S1). Consequently, the MetaSubtract method^41^ was applied to the BP, MDD and PTSD GWASs to remove overlapping FinnGen samples from the discovery statistics. The same SVD‐based procedures were then repeated to recompute the PCs with the same 2259 SNPs, and PC‐PGSs were subsequently calculated in FinnGen samples. For each disorder, a PGS corresponding to each PC was calculated. These PC‐PGSs were defined as the sum of effect alleles weighted by their contribution to the corresponding PC, as previously described.^21^ To enable PGS calculation from summary statistics, the group‐wise polygenic risk score (GrpPRS) module implemented in the Reconstructing Allelic Count (ReACt) tool was used.^42^ This tool estimates mean PC‐PGS and standard deviation for cases and controls from GWAS summary statistics and tests for differences using a two‐sample *t*‐test.

In addition, because PC6, PC7 and PC8 were hypothesised to reflect dimensions relevant to the differential diagnosis between ASD and ADHD, SCZ and BP, and MDD and PTSD, respectively, case–case GWASs (ccGWASs) were conducted for each of these disorder pairs using the latest FinnGen summary statistics and the ccGWAS module of ReACt. Subsequently, mean PC‐PGS and standard deviations were estimated for these three PCs in both groups of cases, and associations were tested using the GrpPRS module of ReACt. The proportion of variance explained (*R* ^2^) was derived from the corresponding *t*‐statistics. Bonferroni correction was applied to account for multiple testing across the three case–case comparisons.

## RESULTS

### Summary statistics and matrix construction

The eight GWASs of psychiatric disorders are summarised in table S1. The excluded GWASs are summarised in table S2. After the application of the filters, 2259 SNPs were retained. All eight psychiatric disorder GWASs contributed non‐zero *Z*‐scores, ranging from 142 SNPs for ASD to 1530 SNPs for MDD.

### Singular value decomposition

**FIGURE 3:**
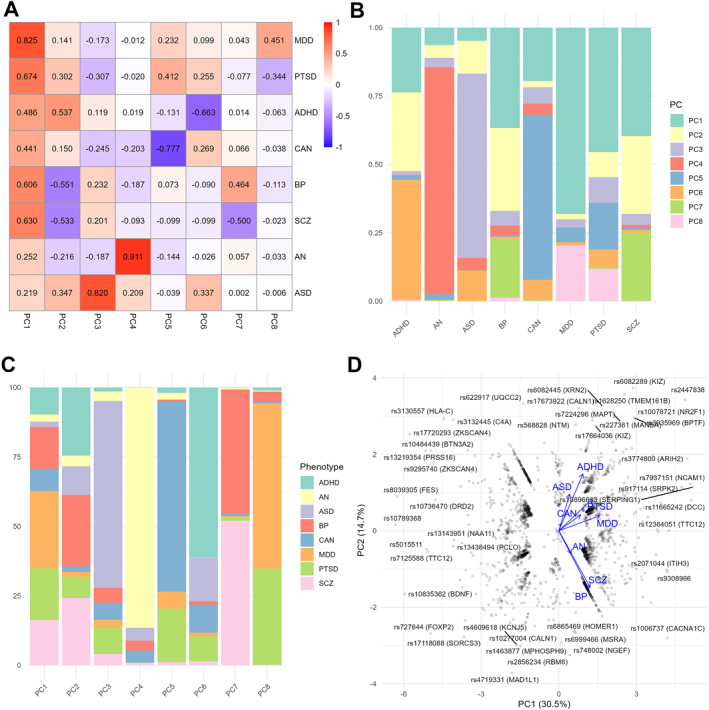
Characterisation of the latent components. (A) Heat map of phenotype singular vectors, summarising the strength of the association between each disorder and each PC. (B) Relative contribution of each PC to each disorder. (C) Relative contribution of each disorder to each PC. (D) Variant PCA biplot for the components PC1 and PC2. The plot highlights the 25 SNPs with the largest contributions to any PC, annotated with their corresponding gene symbols, according to the top *otargen* V2G score. Blue biplot arrows indicate the phenotypes used to interpret component directions. ADHD, attention deficit hyperactivity disorder; AN, anorexia nervosa; ASD, autism spectrum disorder; BP, bipolar disorder; CAN, cannabis dependence; MDD, major depressive disorder; PC, principal component; PCA, principal component analysis; PTSD, post‐traumatic stress disorder; SCZ, schizophrenia; SNP, single nucleotide polymorphism; V2G, variant‐to‐gene.

Latent components were identified and characterised by applying SVD to the matrix of *Z*‐scores derived from 2259 SNPs across eight psychiatric disorders (figure 3A–C).

PC1 accounted for 30.5% of the total variance, with all disorders scoring in the same direction (figure 3D). However, the relative contributions of ASD and AN were limited (2.0% and 2.6%, respectively), likely reflecting the smaller sample sizes of their GWASs. Thus, this first component may reflect a general liability to psychopathology. PC2 explained 14.7% of the variance and was characterised by contrasting scores, with thought disorders scoring in the opposite direction to the remaining disorders, with childhood‐onset neurodevelopmental disorders at the other edge (figure 3D).

PC5 (11.0% of the variance) showed opposite sign scores between internalising disorders on one side and the externalising disorders CAN and, to a lesser extent, ADHD on the other. Additional components distinguished closely related diagnostic categories. PC6 (8.9%) was characterised by opposite sign scores for ASD and ADHD; PC7 (6.0%) differentiated between SCZ and BP; and PC8 (4.3%) between MDD and PTSD. These components may capture latent dimensions relevant to differential diagnosis. Several components exhibited strong disorder‐specific contributions, most notably PC4 (AN: 86.5%), followed by PC5 (CAN: 68.4%) and PC3 (ASD: 67.2%). With the exception of AN and ASD, all disorders contributed consistently to at least two PCs (figure 3B).

Most of the PCs remained consistent with those from the original matrix construction across secondary analyses, including the use of different *p* value thresholds, the exclusion of the MHC region and the use of earlier, smaller GWAS releases for SCZ, BP and MDD (figures S1–S4). For example, all psychiatric disorders scored in the same direction on PC1. PC2 contrasted thought disorders with childhood‐onset neurodevelopmental disorders. PC6, PC7 and PC8 also displayed similar patterns as in the primary analysis, contrasting ASD with ADHD, SCZ with BP and MDD with PTSD, respectively.

### Top contributing SNPs

Table S3 presents the contribution of the 2259 SNPs across the eight PCs. The distribution of the cumulative contribution of SNPs to each PC was highly variable (figure S5).

A total of 179 SNPs were identified as contributing to the first 10% of the variance explained in any of the eight PCs, including 16 SNPs contributing to 2 PCs and 4 SNPs contributing to 3 PCs (table S4). These SNPs were rs7937151 and rs3774800, intronic SNPs at *NCAM1* and *ARIH2*, respectively, and SNPs rs2447838 and rs12202969, located at regions devoid of protein‐coding genes. This is also the case for the top contributing SNP to PC1, rs10789368, located within the long noncoding RNA gene *LINC01360*.

### Gene set enrichment analysis

All PCs except PC3 and PC5 showed enrichment in at least one GO biological process at FDR < 0.05 (table S5 and figure S6). All PCs with at least one significant GO term were enriched in neurodevelopmental processes, except for PC7, which was specifically associated with the gamma‐aminobutyric acid signalling pathway and antigen processing and presentation. All PCs were enriched in synaptic organisation, except for PC4, which, among others, was enriched in gland development. Using a 25% cumulative variance threshold, 38 of the 55 gene sets enriched at FDR < 0.05 under the 50% threshold remained nominally significant (*p* value < 0.05), indicating that the primary biological signals were largely preserved.

No enrichment analysis in gene expression among developmental periods in FUMA surpassed the significance threshold after multiple testing correction (FDR) (figures S7–S14). Nominally, PC3 was associated with early prenatal downregulation (hypergeometric test *p* value = 0.030), whereas PC7 and PC8 were associated with early infancy downregulation (hypergeometric test *p* values = 0.007 and 0.021, respectively).

### Correlations with external GWASs

**FIGURE 4:**
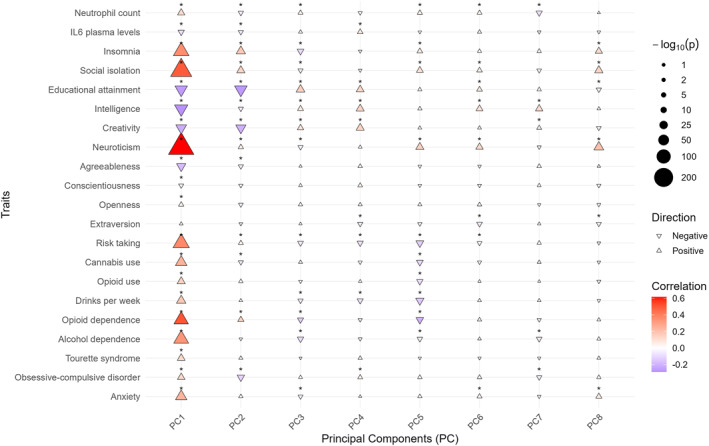
Pearson correlations between PCs and external phenotypes using GWAS summary statistics. Each triangle represents a correlation between a PC (*x*‐axis) and an external phenotype (*y*‐axis), with colour indicating the correlation strength, the triangle orientation indicating the direction of the correlation and triangle size reflecting statistical significance. *FDR < 0.05. FDR, false discovery rate; GWAS, genome‐wide association study; PCs, principal components.

Eighty‐two of the 168 correlations were significant at FDR < 0.05. All the PCs showed some significant correlation with the analysed traits and vice versa (figure 4, table S6). PC1 was significantly correlated with all traits except extraversion, showing the strongest correlation with each correlated trait among the different PCs. The strongest correlations for each PC were PC1—neuroticism (*r* = 0.615; adjusted *p* value < 0.001); PC2—educational attainment (*r* = −0.268; adjusted *p* value < 0.001); PC3—educational attainment (*r* = 0.162; adjusted *p* value < 0.001); PC4—creativity (*r* = 0.142; adjusted *p* value < 0.001); PC5—opioid dependence (*r* = −0.207; adjusted *p* value < 0.001); PC6—neuroticism (*r* = 0.129; adjusted *p* value < 0.001); PC7—intelligence (*r* = 0.122; adjusted *p* value < 0.001); and PC8—neuroticism (*r* = 0.205; adjusted *p* value < 0.001). All psychiatric disorders not included in the SVD matrix were positively genetically correlated with PC1. Substance use traits and risk‐taking behaviour were positively genetically correlated with PC1 and negatively genetically correlated with PC5, in agreement with the negative loading of CAN in PC5. Educational attainment, intelligence and creativity showed a similar pattern of correlations.

### PGSs in FinnGen samples

**FIGURE 5:**
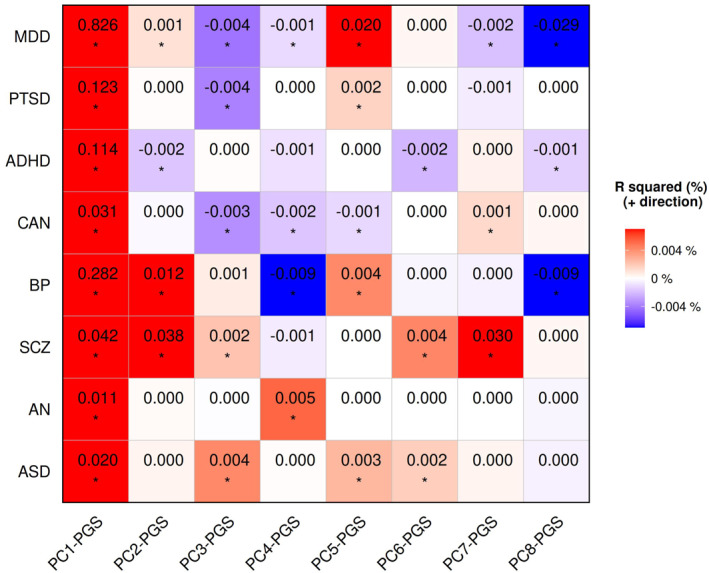
Association between component‐based polygenic scores (PC‐PGS; *x*‐axis) and psychiatric disorders (*y*‐axis) in FinnGen release DF12. The heat map displays the direction and strength of association based on *R* ^2^ values converted from *t*‐test statistics. Colours indicate the direction of the effect, derived from the difference in mean PC‐PGS values between cases and controls, whereas the intensity reflects the proportion of variance explained. *FDR < 0.05. ADHD, attention deficit hyperactivity disorder; AN, anorexia nervosa; ASD, autism spectrum disorder; BP, bipolar disorder; CAN, cannabis dependence; FDR, false discovery rate; MDD, major depressive disorder; PC, principal component; PC‐PGSs, principal component‐based polygenic scores; PTSD, post‐traumatic stress disorder; SCZ, schizophrenia.

All PCs remained largely consistent with those derived from the original matrix after excluding FinnGen data (figure S15). The association between PC‐PGSs and the eight psychiatric disorders in FinnGen was evaluated. In total, 35 associations survived multiple‐testing correction (FDR < 0.05) (figure 5 and table S7). Specifically, PC1‐PGS was found to be significantly associated with all eight psychiatric disorders in FinnGen in the expected direction. PC2‐PGS showed significant associations with BP, SCZ and ADHD in the expected direction. Similar consistency was observed for other combinations, including PC3‐PGS with ASD, PC4‐PGS with AN, PC5‐PGS with CAN, MDD and PTSD, PC6‐PGS with ASD and ADHD, PC7‐PGS with SCZ and PC8‐PGS with MDD, among others.

To evaluate the potential utility of PC6, PC7 and PC8 in the differential diagnosis of ASD versus ADHD, SCZ versus BP and MDD versus PTSD, respectively, PC‐PGSs were calculated using the corresponding ccGWAS summary statistics as the target data. Each PC‐PGS showed a statistically significant association with its corresponding ccGWAS in the expected direction after multiple testing correction (table 1).

**TABLE 1:** PC‐PGS analysis in ccGWAS results using the ReACt framework

Discovery	Target	Mean PGS	SE (mean PGS)	*R* ^2^ ^a^	*p* value
PC6 without FinnGen	ASD versus ADHD ccGWAS	ASD: 1.20 × 10^−4^	ASD: 1.28 × 10^−4^	0.243%	< 0.001^b^
		ADHD: 1.03 × 10^−4^	ADHD: 1.28 × 10^−4^		
PC7 without FinnGen	SCZ versus BP ccGWAS	SCZ: 1.40 × 10^−4^	SCZ: 1.06 × 10^−4^	0.735%	< 0.001^b^
		BP: 1.21 × 10^−4^	BP: 1.06 × 10^−4^		
PC8 without FinnGen	MDD versus PTSD ccGWAS	MDD: 1.87 × 10^−4^	MDD: 1.33 × 10^−4^	0.012%	0.006^b^
		PTSD: 1.94 × 10^−4^	PTSD: 1.33 × 10^−4^		

Abbreviations: ADHD, attention deficit hyperactivity disorder; ASD, autism spectrum disorder; BP, bipolar disorder; ccGWAS, case–case genome‐wide association study; MDD, major depressive disorder; PC‐PGS, polygenic score based on principal component; PTSD, post‐traumatic stress disorder; ReACt, Reconstructing Allelic Count; SCZ, schizophrenia.

^a^ *R* ^2^ corresponds to the regression *R* ^2^ with only the PGS predictor.

^b^ *p* values were obtained from two‐sample *t*‐tests comparing mean PC‐PGSs between case groups. Bonferroni correction was applied for three independent case–case comparisons (*α* = 0.05/3 = 0.017).

## DISCUSSION

### Main findings

To the best of our knowledge, this is the first study to apply SVD to GWAS data of psychiatric disorders. Through this approach, eight biologically coherent and orthogonal PCs were identified. These PCs were characterised by using multiple complementary strategies, including SNP to gene mapping, GO enrichment analysis, Pearson correlation with external traits not included in the original matrix and PGS analyses in the independent FinnGen samples. Twenty SNPs are among the top contributors for more than one PC, consistent with the notion that the varying effect sizes and directions of many pleiotropic SNPs influence which specific disorder manifests.^2^

PC1 recapitulates a previously described polygenic *p* factor using a hypothesis‐free SVD‐based decomposition of GWAS summary statistics.^43^, ^44^, ^45^, ^46^ All psychiatric disorders included in the analysis scored in the same direction on this component, as did the other psychiatric traits in the external correlation analysis. Moreover, PC1‐PGSs were significantly associated with all psychiatric disorders in FinnGen samples. GO enrichment analysis revealed that PC1 was mainly associated with neurodevelopment, in line with previous cross‐disorder findings.^47^ However, this result lacks specificity, as most PCs showed some degree of enrichment in neurodevelopmental processes. The *p* factor has been previously interpreted as related to traits such as neuroticism, impulsivity, cognitive impairment, thought disorder and overall functional decline.^48^, ^49^ Our results point to an important role of neuroticism in the conceptualisation of the *p* factor. Thus, it was the external trait most strongly correlated with PC1 (*r* = 0.615). Other relevant traits in this context presented a considerable but clearly lower correlation (*r* = 0.491 for social isolation, *r* = 0.371 for risk‐taking, *r* = −0.289 for intelligence). These traits may be involved in the same pathway, in agreement with previous literature suggesting that the *p* factor may reflect a general tendency to react impulsively to negative emotional states (neuroticism), ultimately leading to functional impairments such as social isolation or academic underachievement.^49^ A strong correlation between the *p* factor and neuroticism has been previously described at the phenotypic level, although the results are highly dependent on the behavioural data used to derive the factors, with the range of correlations from 0.13 to 0.88.^50^

The top contributing SNP to PC1 was located within *LINC01360*, a long noncoding RNA. Genome editing in the human glioma cell line U251 revealed that increased expression of *LINC01360* led to 34 differentially expressed genes at an adjusted *p* value < 0.001 and fold change > 1.2. One of these genes was *DRD2*.^51^ Interestingly, our SNP‐to‐gene approach identified *DRD2* as the most likely causal gene for rs10736470, one of the top contributing SNPs at this PC. The second most contributing SNP was located within an intron of *FURIN*. This gene codes for a proprotein convertase involved in the proteolysis of several inactive precursors to generate functionally active secretory proteins. Interestingly, the third most contributing SNP to PC1, rs10835362, maps to *BDNF*, one of the substrates of *FURIN*.^52^ A SCZ risk SNP causes reduction of mature BDNF production by downregulation of *FURIN* expression in cell cultures, suggesting that this may be a mechanism involved in the risk for psychiatric disorders.^53^ Thus, SVD of GWAS summary statistics of psychiatric disorders may identify functionally related genes.

PC2 separates disorders involving alterations in thought content, such as SCZ, BP and, to a lesser extent, AN, from other disorders, with childhood‐onset neurodevelopmental disorders at the other edge. In agreement with this, four genes assigned to top contributing SNPs at PC2 (*MAD1L1*, *MPHOSPH9*, *MSRA* and *CALN1*) were significant in previous ccGWASs comparing SCZ versus MDD, ASD or ADHD. One additional gene, *HOMER1*, was significant in a BP versus ADHD GWAS.^54^ Notably, OCD, another condition with symptoms involving thought content (i.e., obsessions), showed a similar correlation pattern. SCZ and BP have been previously associated with creativity at the genetic level,^55^, ^56^ and interestingly, creativity was one of the traits most correlated with PC2, suggesting that this component may capture a propensity for divergent thinking. PGSs derived from this component were associated with SCZ and BP in FinnGen samples in the expected directions, indicating that PC2‐PGS may hold promise for quantifying genetic risk for severe thought disorders. A similar approach could be valuable in precision psychiatry to support more informed clinical research frameworks, although further validation in individual‐level and longitudinal settings is required. For instance, refinement of this latent factor could help identify individuals with treatment‐resistant MDD who are more likely to benefit from antipsychotic augmentation due to its association with thought disorder liability. Conversely, those with lower PC2‐PGS scores may respond better to alternative interventions.^57^

PC3 was primarily linked to ASD risk, although most of the other disorders also contributed to this PC. Among them, BP and SCZ align with the direction of ASD, whereas PTSD, CAN and MDD scored in the other direction. In agreement with this, *XRN2*, one of the mapped genes at the top contributing SNPs for this PC, was significant in an ASD versus MDD GWAS.^54^ PC3 may distinguish highly heritable psychiatric disorders from those more strongly influenced by environmental factors, such as CAN, PTSD and MDD. Therefore, it would be of interest to examine the interaction between PC3‐PGS and key environmental exposures, such as childhood trauma or substance abuse.

PC4 was primarily driven by AN. In addition to neurodevelopmental processes, it was enriched in GO terms related to gland development, highlighting the endocrine‐psychiatric nature of eating disorders.^27^

PC5 was predominantly influenced by CAN. Notably, all substance use‐related traits correlated in the same direction, suggesting that this component may capture a general liability towards substance use disorders.

PC6 was largely defined by ADHD and showed an opposite score direction to ASD, indicating its potential utility in differentiating between these two neurodevelopmental disorders. Remarkably, the PC6‐PGS was associated with both ASD and ADHD in opposite directions in the FinnGen samples and also distinguished between the two disorders in our case–case analysis. Although core symptoms differentiate between the two disorders, comorbidity between them is common, and the use of methylphenidate is frequent in patients diagnosed with ASD. However, the rationale for this additional treatment is sometimes unclear.^58^ A tool such as PC6‐PGS could potentially aid in personalising treatment decisions in such scenarios. Nevertheless, the very low proportion of variance explained limits this application.

PC7 may help distinguish SCZ from BP. This represents a relevant clinical need, given the high diagnostic instability between these two disorders.^59^, ^60^ Although the PC7‐PGS was associated with SCZ (vs. controls) but not with BP in the FinnGen samples, our case–case analysis revealed a significant association. This component was enriched in GABAergic signalling and immune‐related pathways, including antigen processing and presentation. Neutrophil count, one of the traits most strongly correlated with PC7, was aligned with the SCZ direction, whereas intelligence was associated with the BP direction. Therefore, in patients experiencing a first episode of psychosis, a tool such as PC7‐PGS analysis could be useful for predicting diagnostic trajectory as well as response to adjunctive treatments.^61^, ^62^

PC8 appeared to differentiate MDD from PTSD. Although the PC8‐PGS was associated only with MDD (vs. controls) in the FinnGen samples and not with PTSD, our case–case analysis showed a significant association. Exposure to traumatic events is a prerequisite for the diagnosis of PTSD and a risk factor for MDD. Therefore, a tool such as PC8‐PGS could aid in predicting the development of MDD or PTSD after a traumatic event, thereby helping to anticipate the use of effective targeted treatments.^63^ It would be of interest to examine potential gene–environment interactions between PC8‐PGS and exposure to traumatic life events.^64^, ^65^

### Limitations

This study has several limitations. First, there was considerable heterogeneity in the sample sizes of the GWASs included. GWASs with smaller sample sizes tended to contribute predominantly to PCs with minimal or no contributions from other disorders. Nevertheless, most of the PCs remained consistent with those from the original matrix construction using earlier, smaller GWAS releases for SCZ, BP and MDD. Second, there may be heterogeneity in the diagnostic definitions across the discovery GWASs. Some GWASs, such as those for SCZ, include patients diagnosed using strict clinical criteria, whereas others, such as MDD, rely on broader definitions. Third, some degree of residual sample overlap across large psychiatric GWASs cannot be fully excluded, as many rely on partially shared biobank resources and control samples. However, several observations argue against sample overlap being the sole driver of PC1: (i) Similar broad components have been consistently reported across cross‐disorder analyses using different methodologies, such as genomic SEM, which is not affected by sample overlap; (ii) PC1 showed coherent biological enrichment and meaningful correlations with external traits not included in the SVD matrix; (iii) PC‐PGS analyses conducted in FinnGen using the GrpPRS framework explicitly excluded sample overlap through the MetaSubtract procedure yet yielded consistent associations. Fourth, PC‐PGSs were evaluated using the ReACt framework, which estimates mean score differences between cases and controls from GWAS summary statistics. As such, these analyses do not assess individual‐level predictive performance or clinical utility and should be interpreted as group‐level associations, pending validation in individual‐level and longitudinal settings. Therefore, their current applicability for clinical risk prediction or decision‐making remains limited. Moreover, even after such validation, PGSs alone are expected to have limited predictive value and will likely be most informative when integrated with other genetic and nongenetic factors.^16^ Fifth, the PC‐PGS analyses did not correct for stratification between cases and controls, as they were based on mean PC‐PGS in cases and controls rather than individual values. However, prior to GWAS analysis, FinnGen removed outlier samples, that is, those with < 95% probability of being part of the Finnish cluster in principal component analysis with other European populations from the 1000 Genomes as a reference. Closely related samples were also removed.^40^ Sixth, the inclusion of additional disorders could alter the resulting pattern of PCs. The selection was limited to eight GWASs to avoid excessively reducing the number of SNPs retained for analysis. Furthermore, the applied threshold of retaining GWASs with more than 20 000 SNPs with *p* values < 0.001 was set based on the data distribution, as the lowest‐ranking included GWAS surpassed this threshold with 29 839 SNPs (ASD), whereas the next GWAS not included had only 15 920 (Tourette syndrome). Seventh, SVD detects orthogonal dominant dimensions of covariance by simple linear combinations of genetic effects. This may be an oversimplification in the case of epistasis between SNPs and correlation between latent factors. There are many different approaches to identifying the latent factors causing pleiotropy.^66^ Some of these alternative approaches could identify different latent factors more closely related to underlying biology. Finally, all datasets analysed were from individuals of European ancestry, which limits the generalisability of the identified PCs and PC‐PGSs to other populations. The stability and reproducibility of these components, as well as the performance of PC‐PGSs, may differ in non‐European samples due to differences in genetic architecture across ancestries, particularly in allele frequencies and LD patterns, as well as different sociocultural contexts. Future work applying this framework to multiancestry GWAS datasets will be essential to assess transferability and to evaluate the impact of population‐specific characteristics.

### Implications

In conclusion, this study demonstrates that applying SVD to GWAS summary statistics can uncover biologically meaningful dimensions of shared genetic risk across psychiatric disorders, which could be clinically relevant. The resulting PCs capture both transdiagnostic and disorder‐specific patterns. Estimation of the polygenic risk conferred by these PCs could help refine diagnosis and guide personalised treatment strategies.^12^ This approach offers a promising framework for advancing psychiatric genetics towards greater clinical utility. Future studies should evaluate the predictive value of PC‐PGSs in longitudinal cohorts to assess their potential for anticipating diagnostic evolution and treatment response.

## AUTHOR CONTRIBUTIONS

Javier Costas conceptualised the study and managed part of the analyses. Fernando Facal and Ana M. Pérez‐Gutiérrez managed part of the analyses. Fernando Facal wrote the first draft of the manuscript. All authors contributed to and have approved the final manuscript. All authors are responsible for the overall content as guarantors.

## FUNDING

This study was supported by grants from the Instituto de Salud Carlos III (ISCIII) (Grant Numbers ISCIII/PI23/00731 and RD24/0003/0016), cofunded by FEDER, to Javier Costas.

## CONFLICT OF INTEREST STATEMENT

The authors declare no conflicts of interest.

## ETHICS STATEMENT

This study did not involve the use of individual‐level data. The GWAS summary statistics used in this study originate from large public consortia, including the Psychiatric Genomics Consortium (PGC; https://pgc.unc.edu), the Million Veteran Program (MVP; https://www.research.va.gov/mvp/) and FinnGen (https://www.finngen.fi). All GWASs included had obtained written informed consent from participants and received approval from the corresponding institutional ethics committees. The research was conducted in accordance with the most recent revision of the Declaration of Helsinki.

## Supporting information

Figures S1–S15

Tables S1–S7

## Data Availability

All data associated with this study are present in the manuscript or supporting information (Figures S1–S15 and Tables S1–S7). The GWAS summary statistics analysed are publicly accessible from the corresponding consortia.
