# Supplementary material for: Dissecting the polygenic architecture of psychopathology via singular value decomposition of eight psychiatric genome‐wide association studies and evaluation of component‐based polygenic scores
Source: Gen Psychiatr. 2026 Jun 1;39(3):e70027. doi: 10.1002/gps3.70027 (PMC13239150; doi:10.1002/gps3.70027)
Supplement: Supplementary file 1 — Figures S1–S15 [file GPS3-39-e70027-s002.pdf]

Supplementary Figures

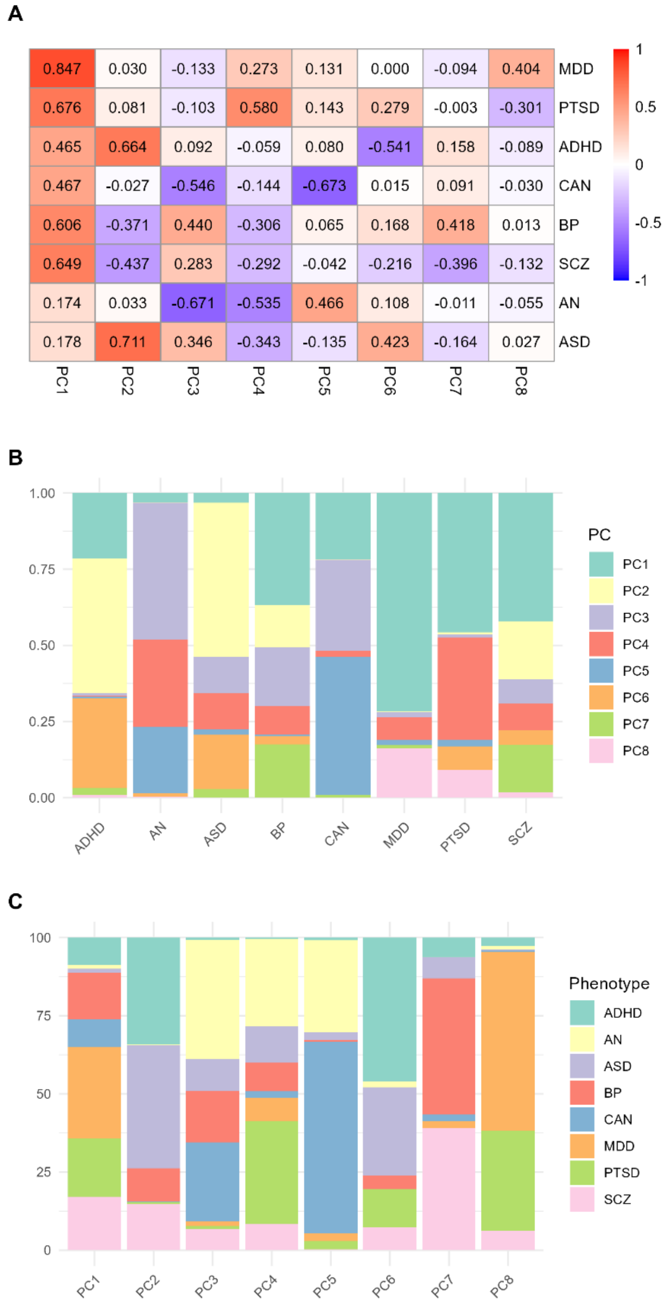

Supplemental figure 1. A) Heatmap of phenotype singular vectors, summarizing the strength of the association between each disorder and each PC, using a *p* value threshold of 1 x 10^{-5} (matrix composed of 313 SNPs). B) Relative contribution of each PC to each disorder. C) Relative contribution of each disorder to each PC.

ADHD, attention deficit hyperactivity disorder; AN, anorexia nervosa; ASD, autism spectrum disorder; BP, bipolar disorder; CAN, cannabis dependence; MDD, major depressive disorder; PC, principal component; PTSD, post-traumatic stress disorder; SCZ, schizophrenia.

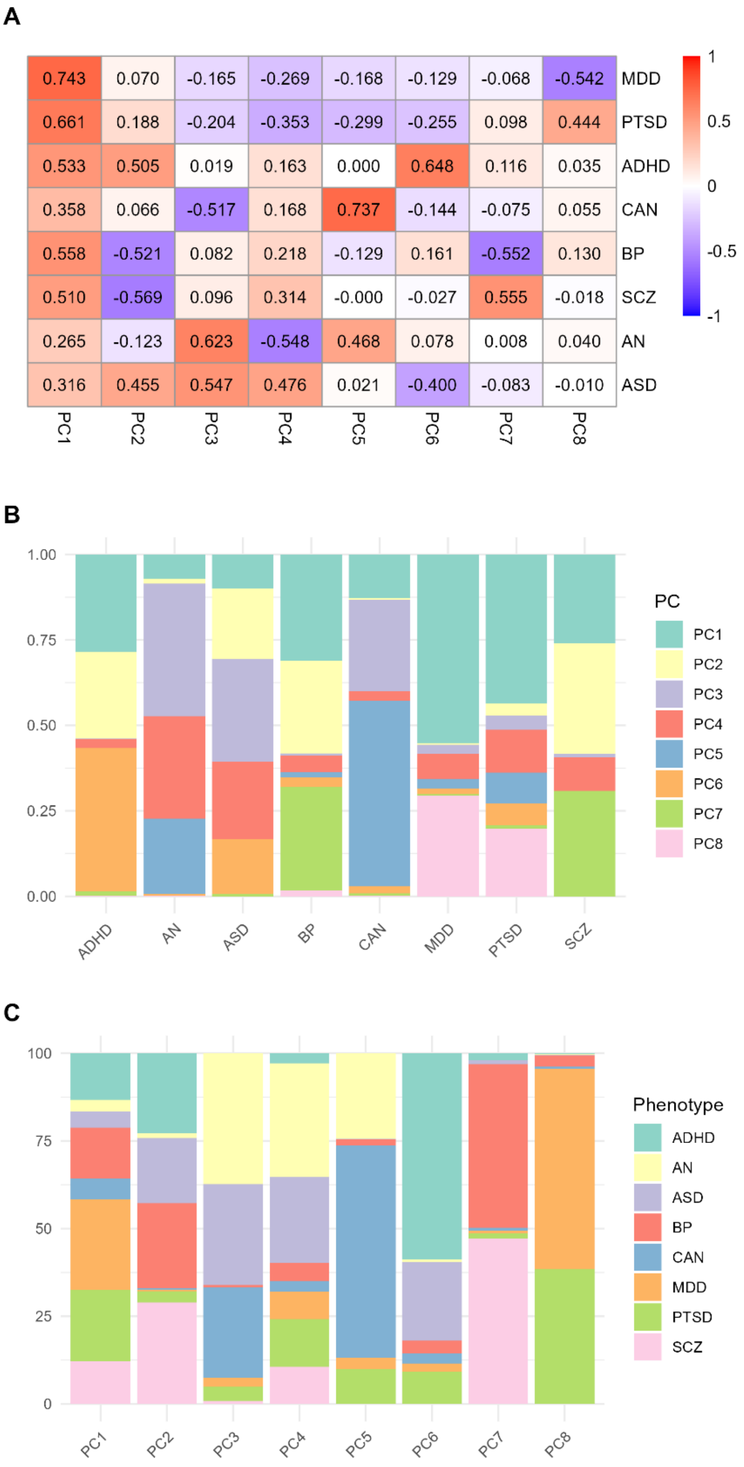

Supplemental figure 2. A) Heatmap of phenotype singular vectors, summarizing the strength of the association between each disorder and each PC, using a *p* value threshold of 0.05 (matrix composed of 39 775 SNPs). B) Relative contribution of each PC to each disorder. C) Relative contribution of each disorder to each PC

ADHD, attention deficit hyperactivity disorder; AN, anorexia nervosa; ASD, autism spectrum disorder; BP, bipolar disorder; CAN, cannabis dependence; MDD, major depressive disorder; PC, principal component; PTSD, post-traumatic stress disorder; SCZ, schizophrenia.

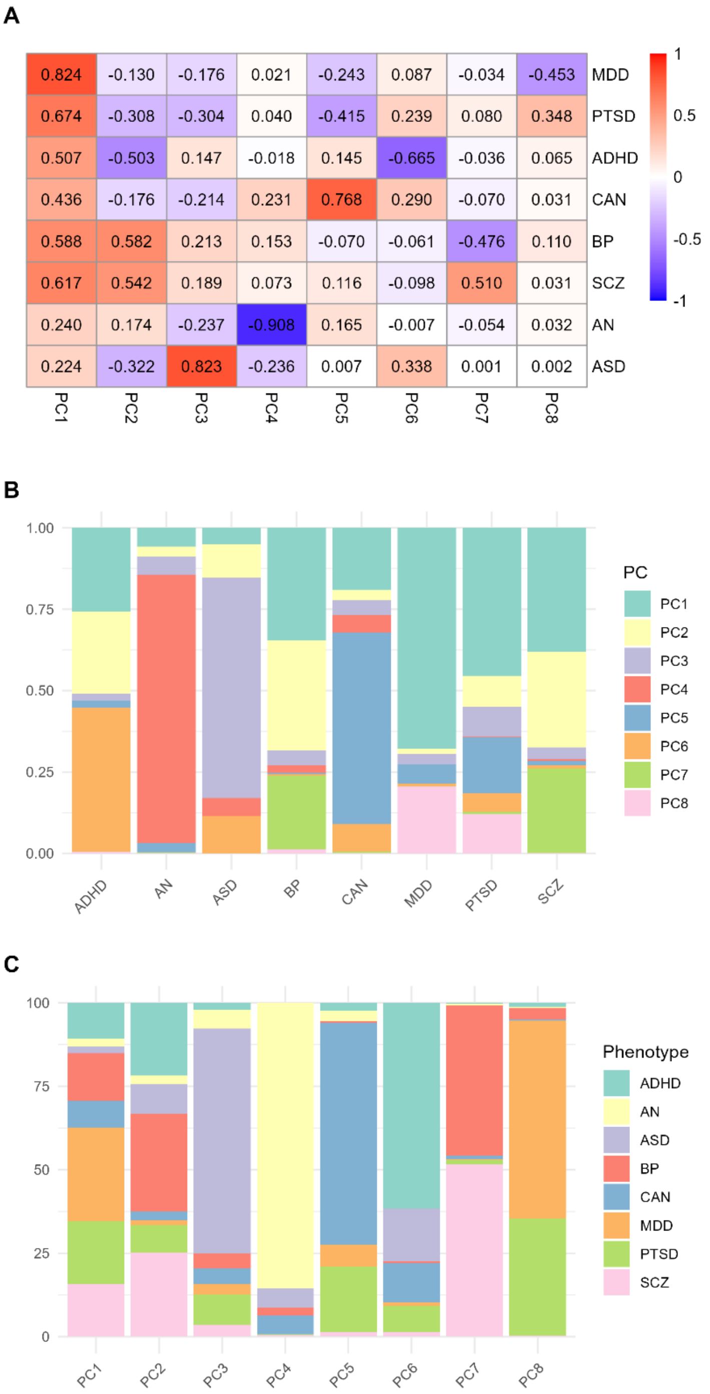

Supplemental figure 3. A) Heatmap of phenotype singular vectors, summarizing the strength of the association between each disorder and each PC, after excluding the major histocompatibility complex (MHC) region (chromosome 6: 25 477 797-36 448 354 base pairs hg19) (matrix composed of 2209 SNPs). B) Relative contribution of each PC to each disorder. C) Relative contribution of each disorder to each PC

ADHD, attention deficit hyperactivity disorder; AN, anorexia nervosa; ASD, autism spectrum disorder; BP, bipolar disorder; CAN, cannabis dependence; MDD, major depressive disorder; PC, principal component; PTSD, post-traumatic stress disorder; SCZ, schizophrenia.

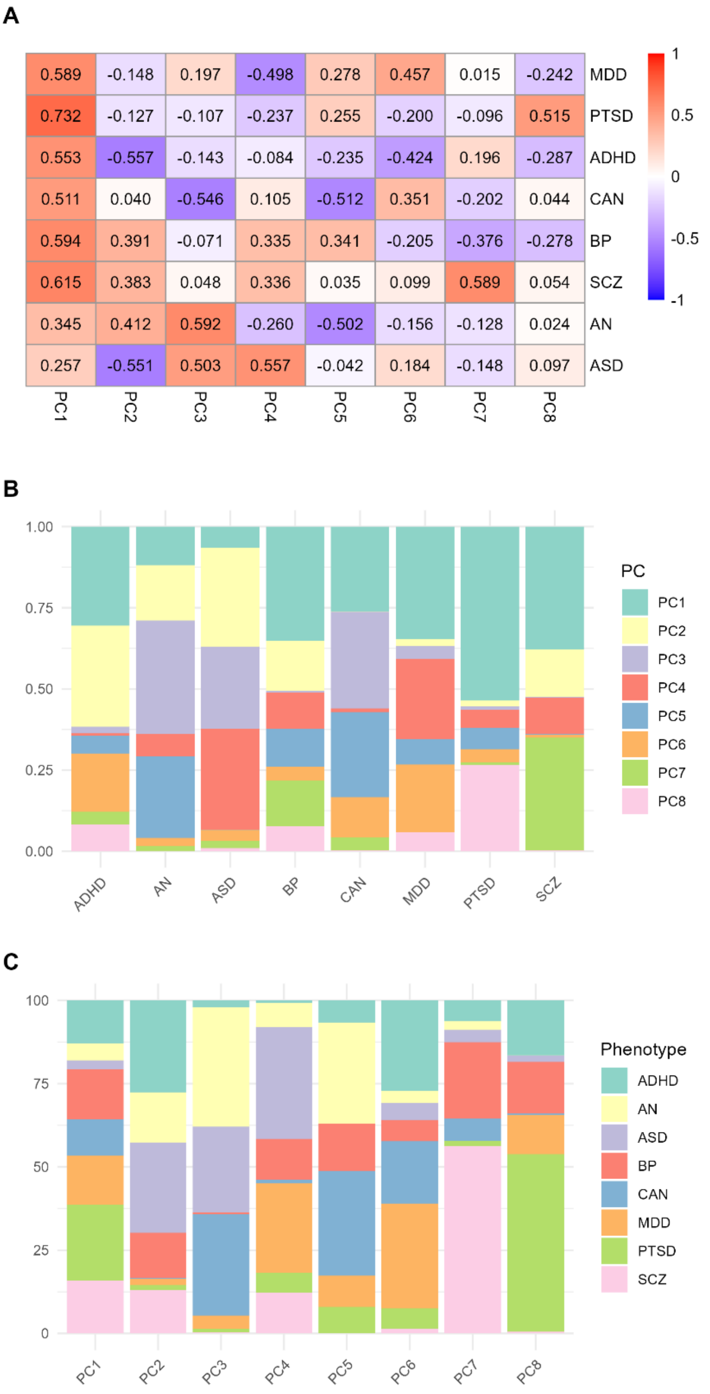

Supplemental figure 4. A) Heatmap of phenotype singular vectors, summarizing the strength of the association between each disorder and each PC, using earlier Psychiatric Genomics Consortium releases with smaller European sample sizes for SCZ (33 640 cases and 43 456 controls); BP (41 917 cases and 371 549 controls); and MDD (59 851 cases and 113 154 controls) (matrix composed of 955 SNPs). B) Relative contribution of each PC to each disorder. C) Relative contribution of each disorder to each PC

ADHD, attention deficit hyperactivity disorder; AN, anorexia nervosa; ASD, autism spectrum disorder; BP, bipolar disorder; CAN, cannabis dependence; MDD, major depressive disorder; PC, principal component; PTSD, post-traumatic stress disorder; SCZ, schizophrenia.

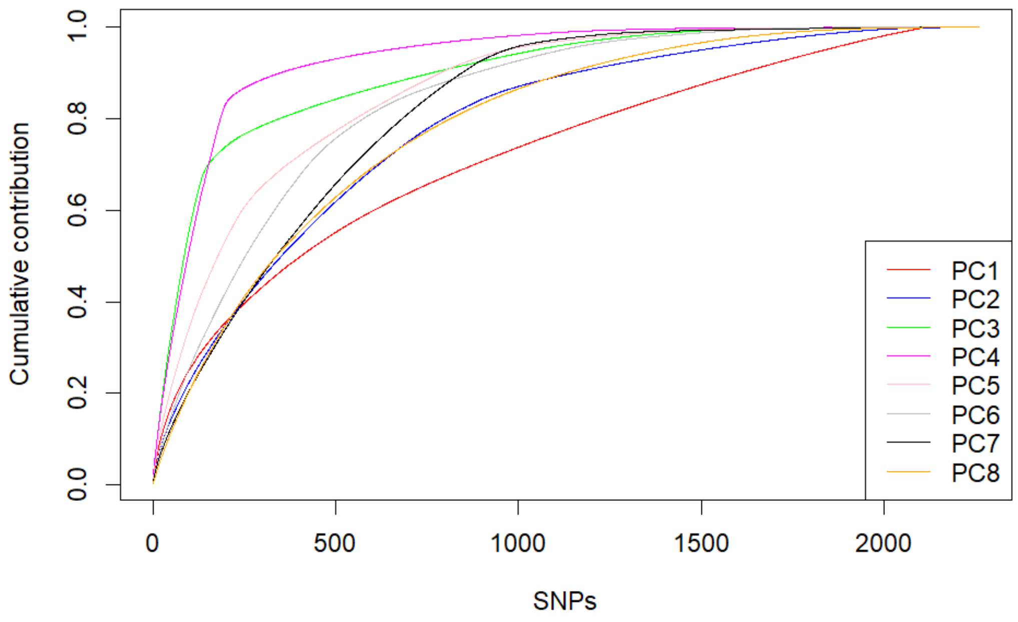

Supplemental figure 5. Cumulative contribution of SNPs to each principal component (PC).

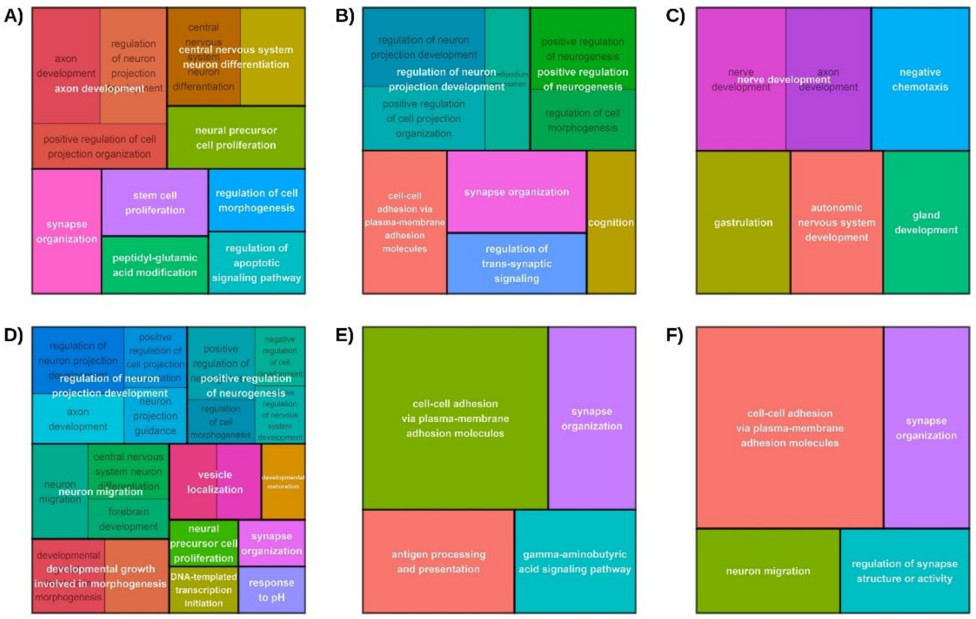

Supplemental figure 6. Treemap plots, generated with the *rrvgo* package in RStudio, of non-redundant Gene Ontology (GO) biological processes for A) PC1, B) PC2, C) PC4, D) PC6, E) PC7 and F) PC8. Significantly enriched pathways (FDR < 0.05) were integrated to highlight higher-level biological processes. Gene-set enrichment was assessed using single nucleotide polymorphisms (SNPs) that cumulatively accounted for 50% of the variance of each PC.

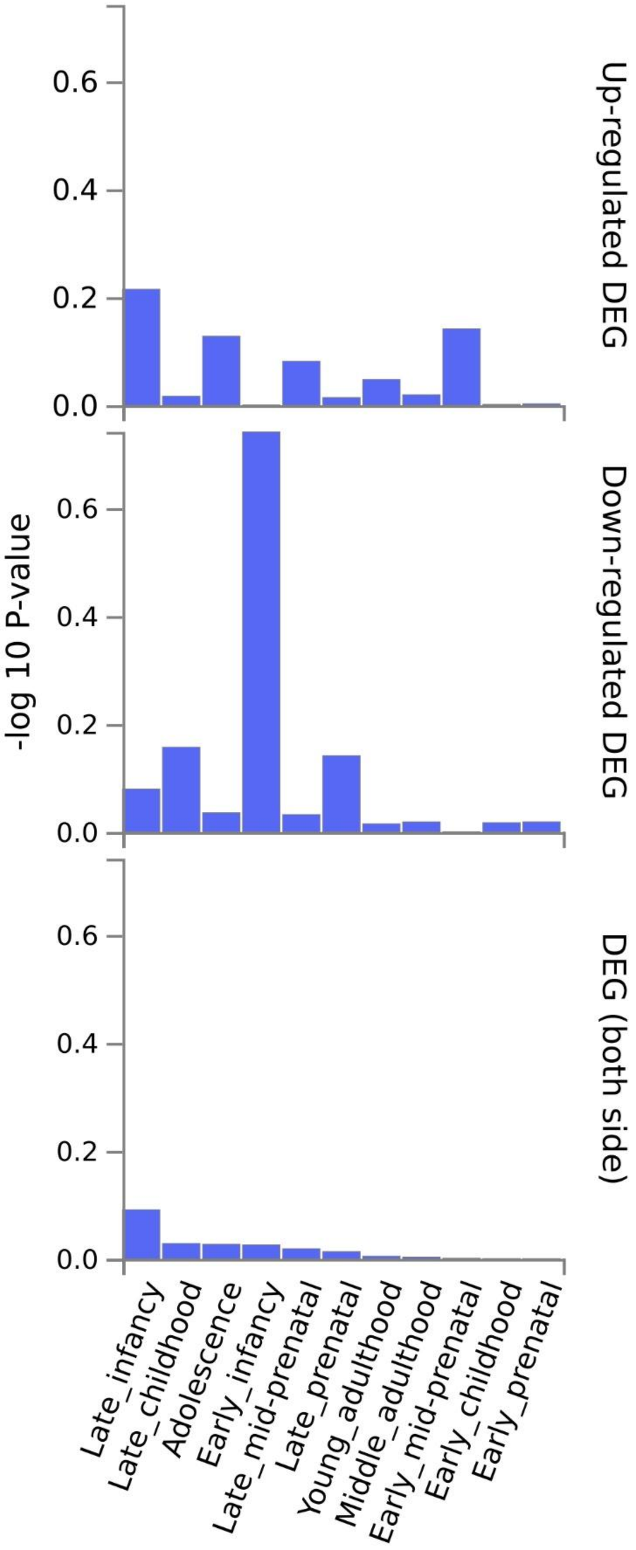

Supplemental figure 7. Differential gene expressions across developmental stages for PC1, based on BrainSpan data using FUMA. Bars indicate the -$\log_{10}$ *p* value for enrichment of genes highly contributing to the component among differentially expressed genes (DEGs) across brain developmental stages. Results are shown separately for upregulated DEGs (top), downregulated DEGs (middle), and both directions combined (bottom).

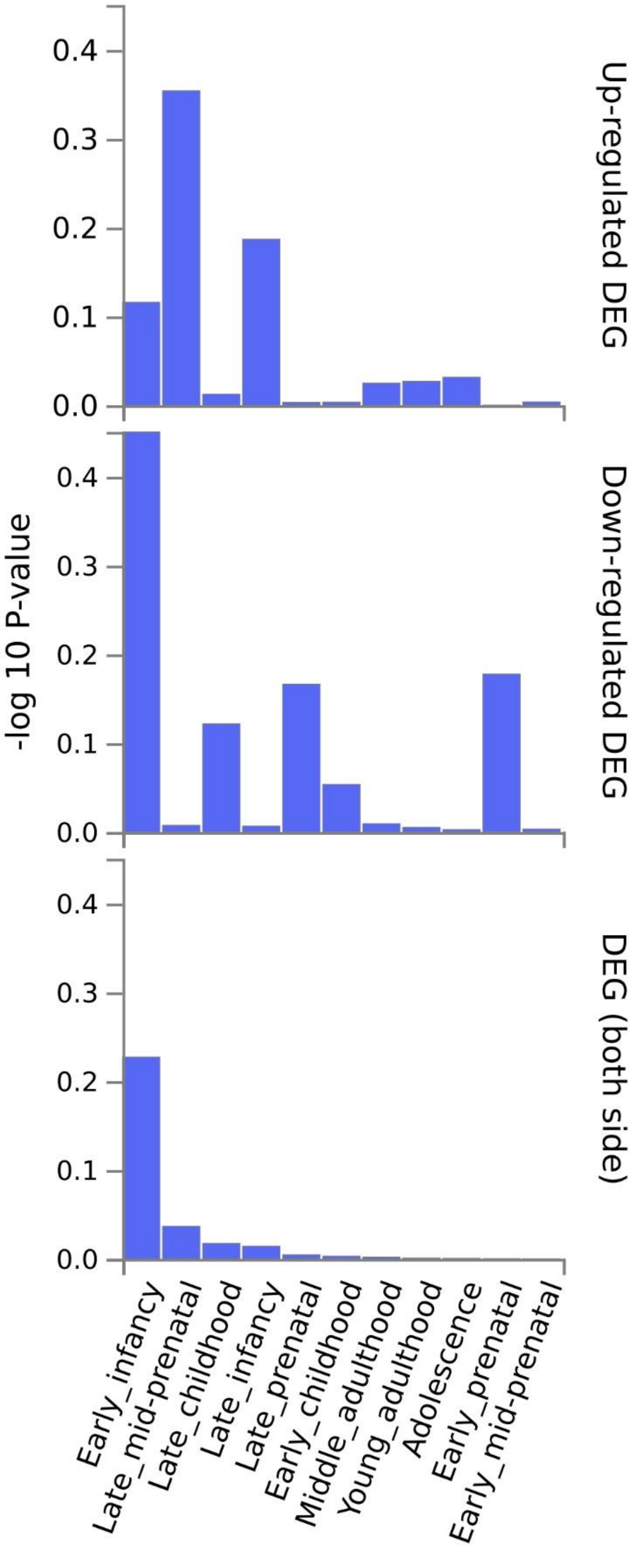

Supplemental figure 8. Differential gene expressions across developmental stages for PC2, based on BrainSpan data using FUMA. Bars indicate the -$\log_{10}$ *p* value for enrichment of genes highly contributing to the component among differentially expressed genes (DEGs) across brain developmental stages. Results are shown separately for upregulated DEGs (top), downregulated DEGs (middle), and both directions combined (bottom).

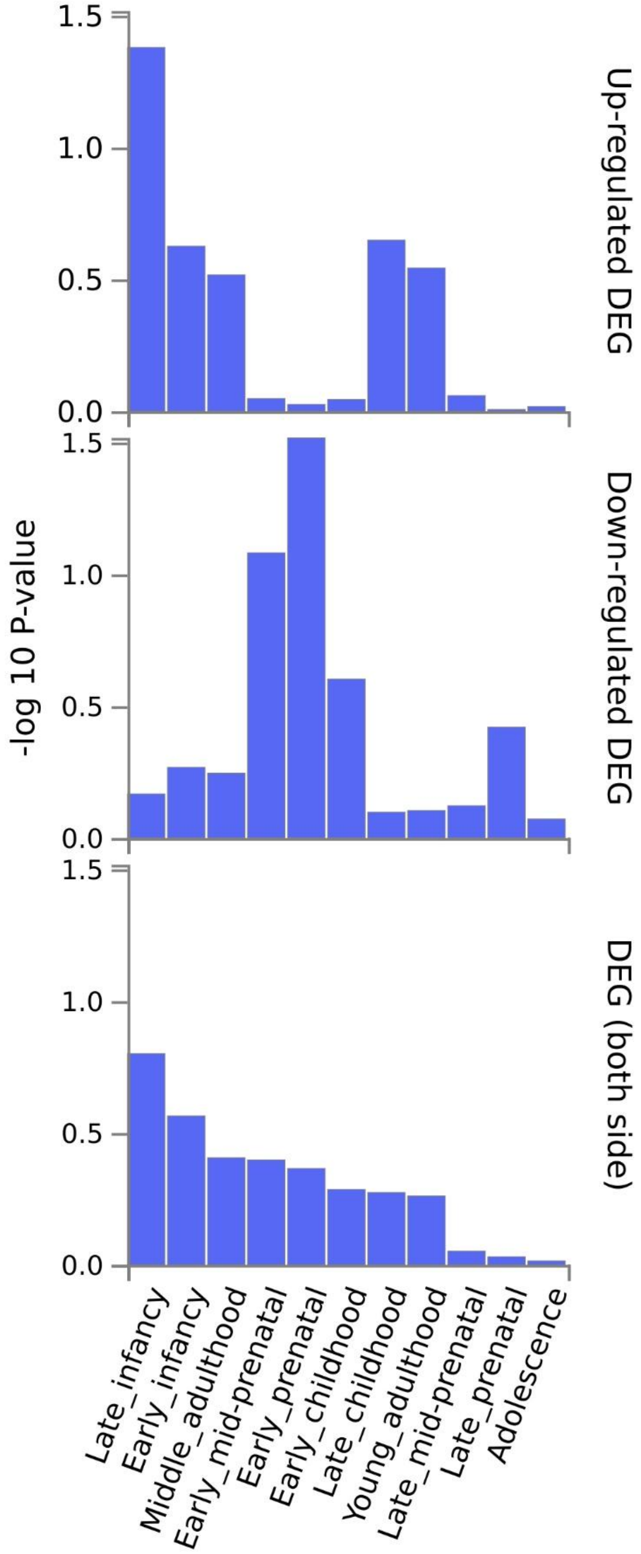

Supplemental figure 9. Differential gene expressions across developmental stages for PC3, based on BrainSpan data using FUMA. Bars indicate the -$\log_{10}$ p value for enrichment of genes highly contributing to the component among differentially expressed genes (DEGs) across brain developmental stages. Results are shown separately for upregulated DEGs (top), downregulated DEGs (middle), and both directions combined (bottom).

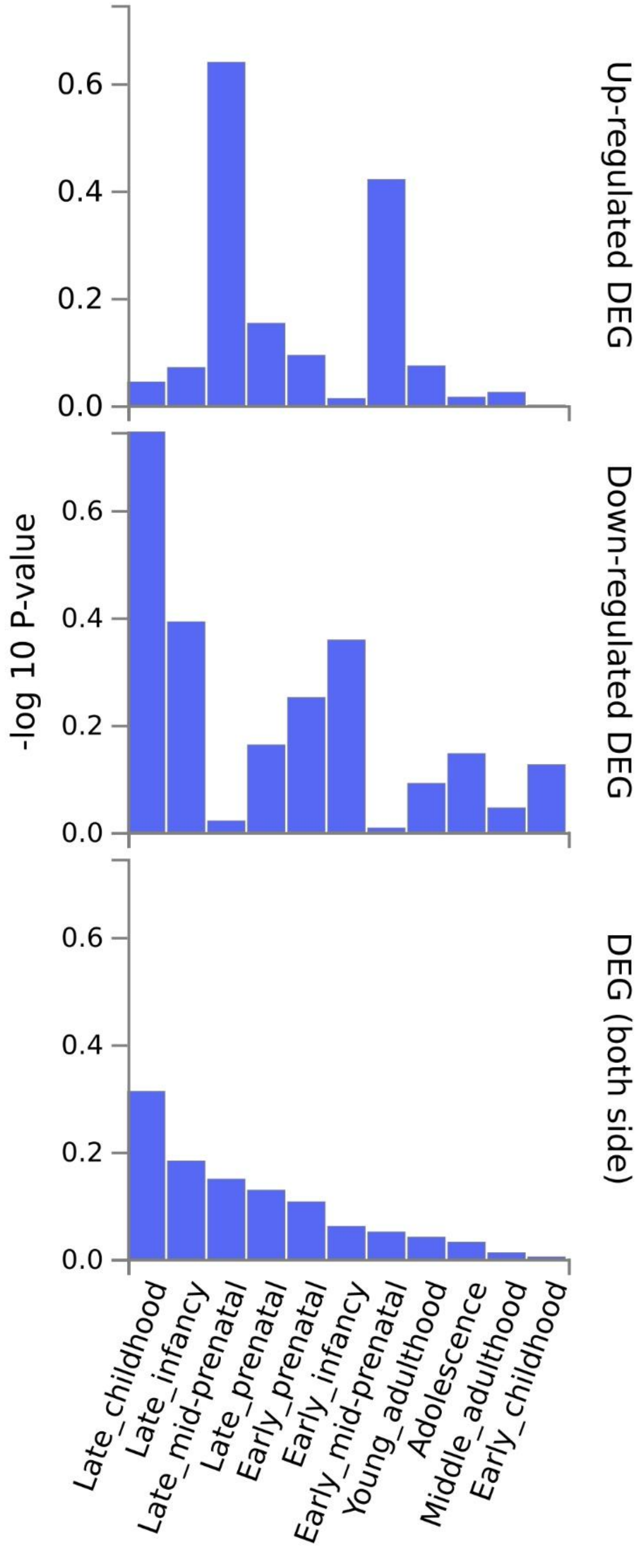

Supplemental figure 10. Differential gene expressions across developmental stages for PC4, based on BrainSpan data using FUMA. Bars indicate the -$\log_{10}$ *p* value for enrichment of genes highly contributing to the component among differentially expressed genes (DEGs) across brain developmental stages. Results are shown separately for upregulated DEGs (top), downregulated DEGs (middle), and both directions combined (bottom).

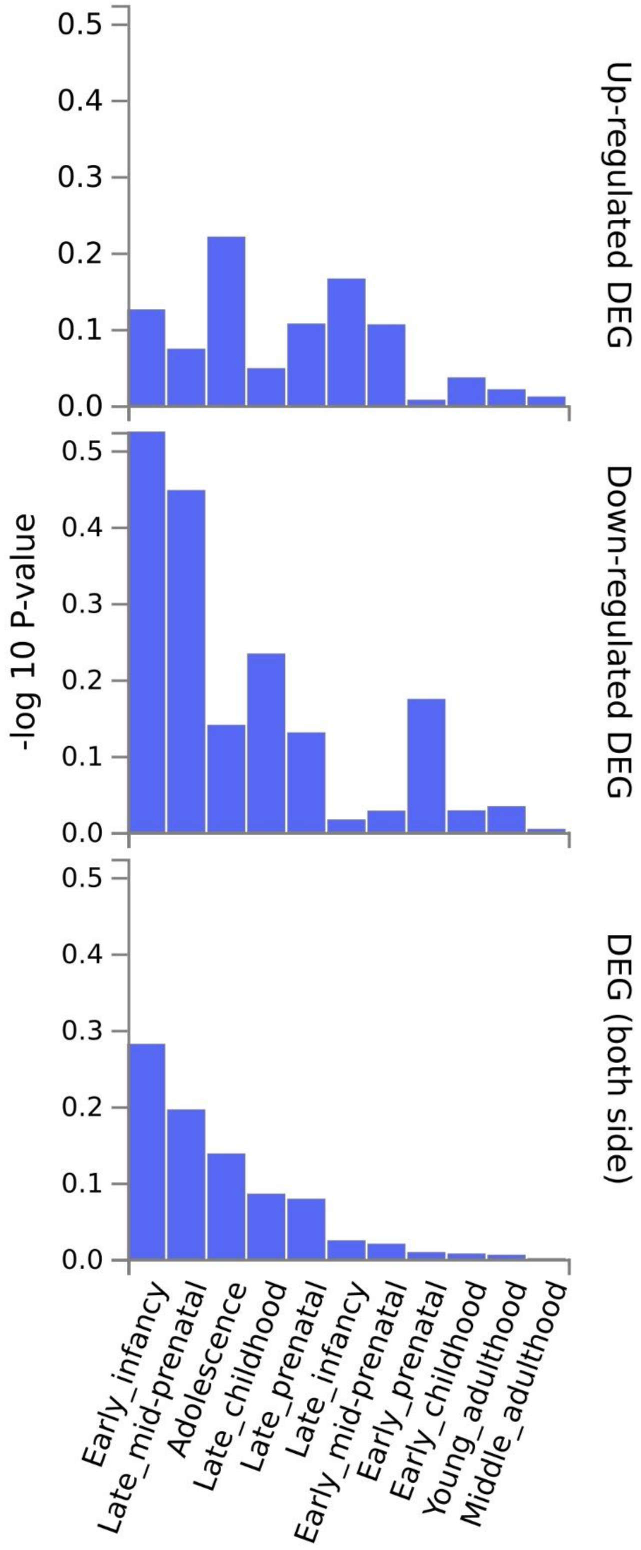

Supplemental figure 11. Differential gene expressions across developmental stages for PC5, based on BrainSpan data using FUMA. Bars indicate the $-\log_{10} p$ value for enrichment of genes highly contributing to the component among differentially expressed genes (DEGs) across brain developmental stages. Results are shown separately for upregulated DEGs (top), downregulated DEGs (middle), and both directions combined (bottom).

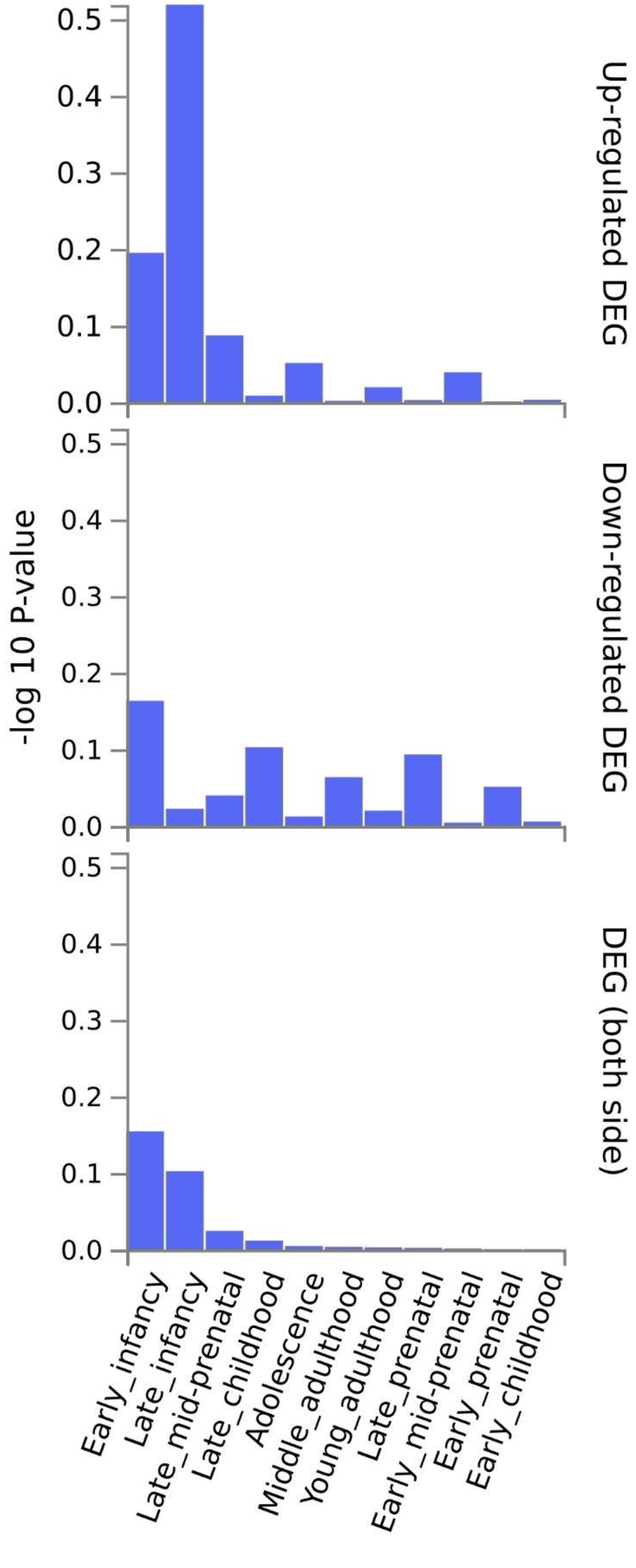

Supplemental figure 12. Differential gene expressions across developmental stages for PC6, based on BrainSpan data using FUMA. Bars indicate the -$\log_{10}$ *p* value for enrichment of genes highly contributing to the component among differentially expressed genes (DEGs) across brain developmental stages. Results are shown separately for upregulated DEGs (top), downregulated DEGs (middle), and both directions combined (bottom).

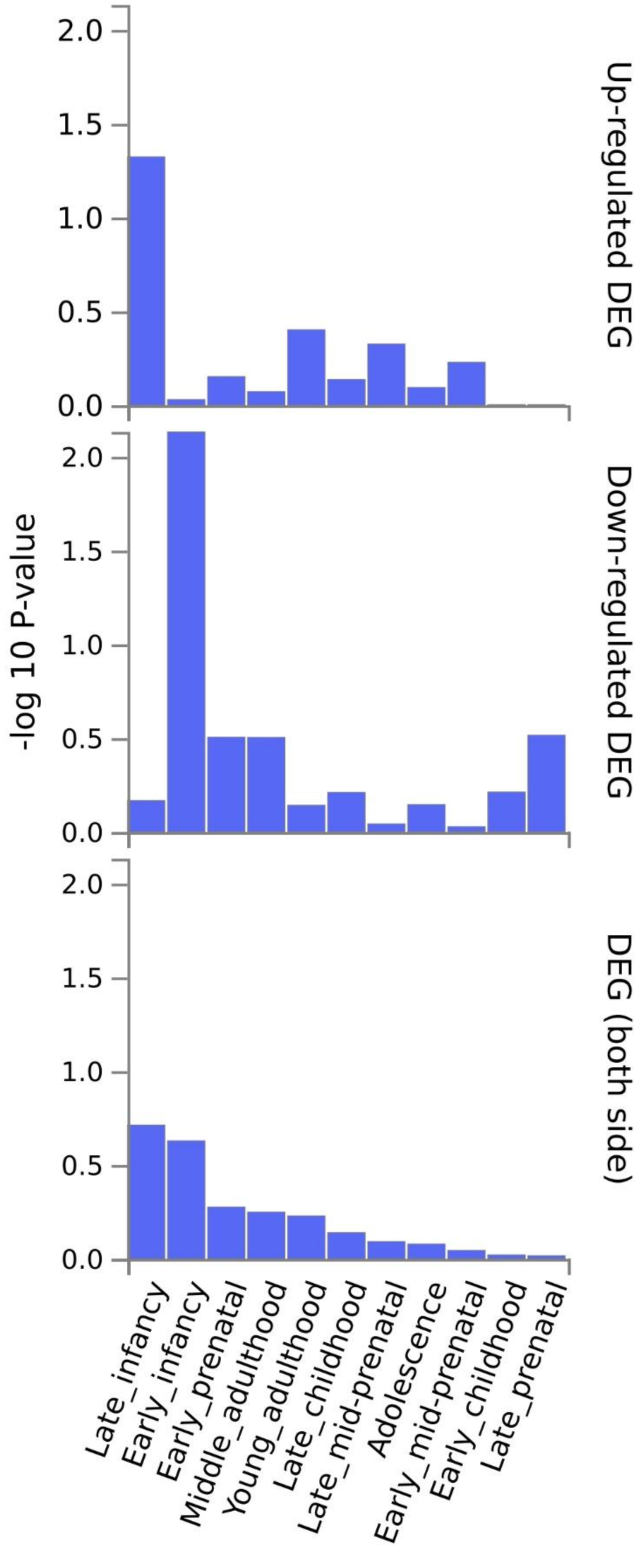

Supplemental figure 13. Differential gene expressions across developmental stages for PC7, based on BrainSpan data using FUMA. Bars indicate the $-\log_{10}$ *p* value for enrichment of genes highly contributing to the component among differentially expressed genes (DEGs) across brain developmental stages. Results are shown separately for upregulated DEGs (top), downregulated DEGs (middle), and both directions combined (bottom).

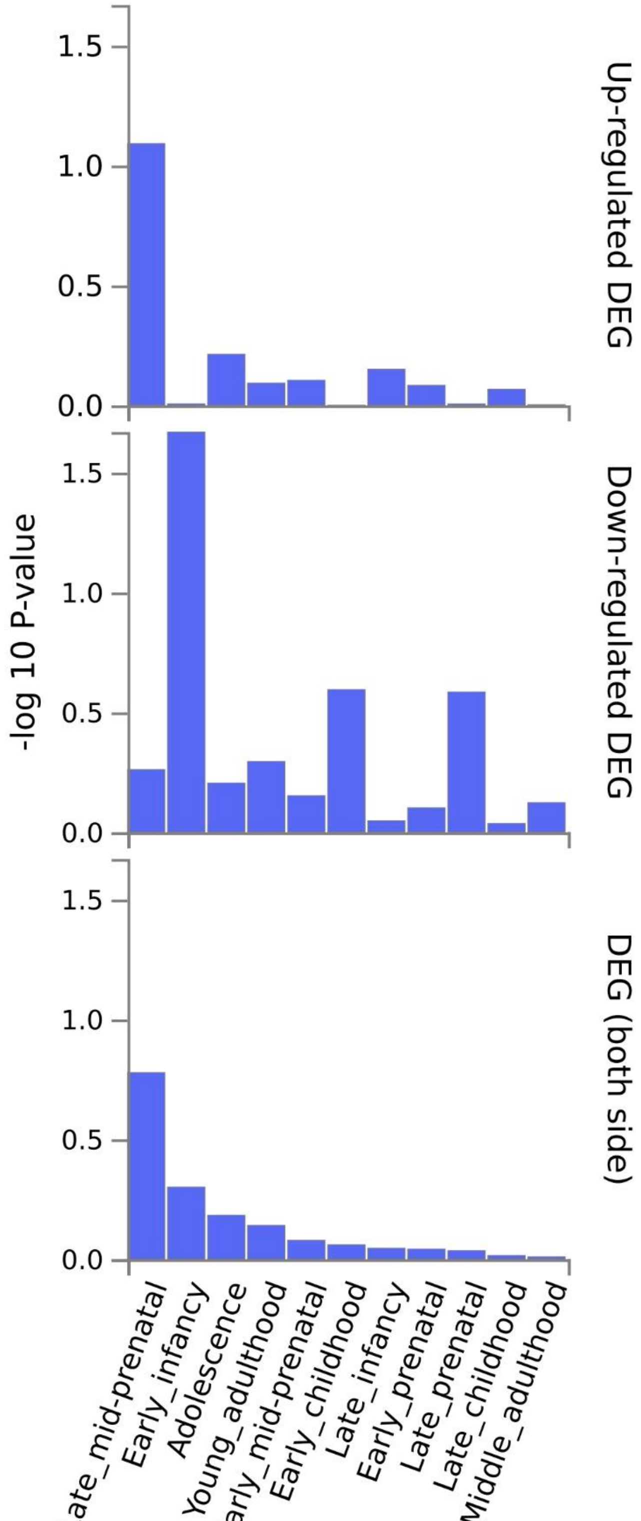

Supplemental figure 14. Differential gene expressions across developmental stages for PC8, based on BrainSpan data using FUMA. Bars indicate the -$\log_{10}$ *p* value for enrichment of genes highly contributing to the component among differentially expressed genes (DEGs) across brain developmental stages. Results are shown separately for upregulated DEGs (top), downregulated DEGs (middle), and both directions combined (bottom).

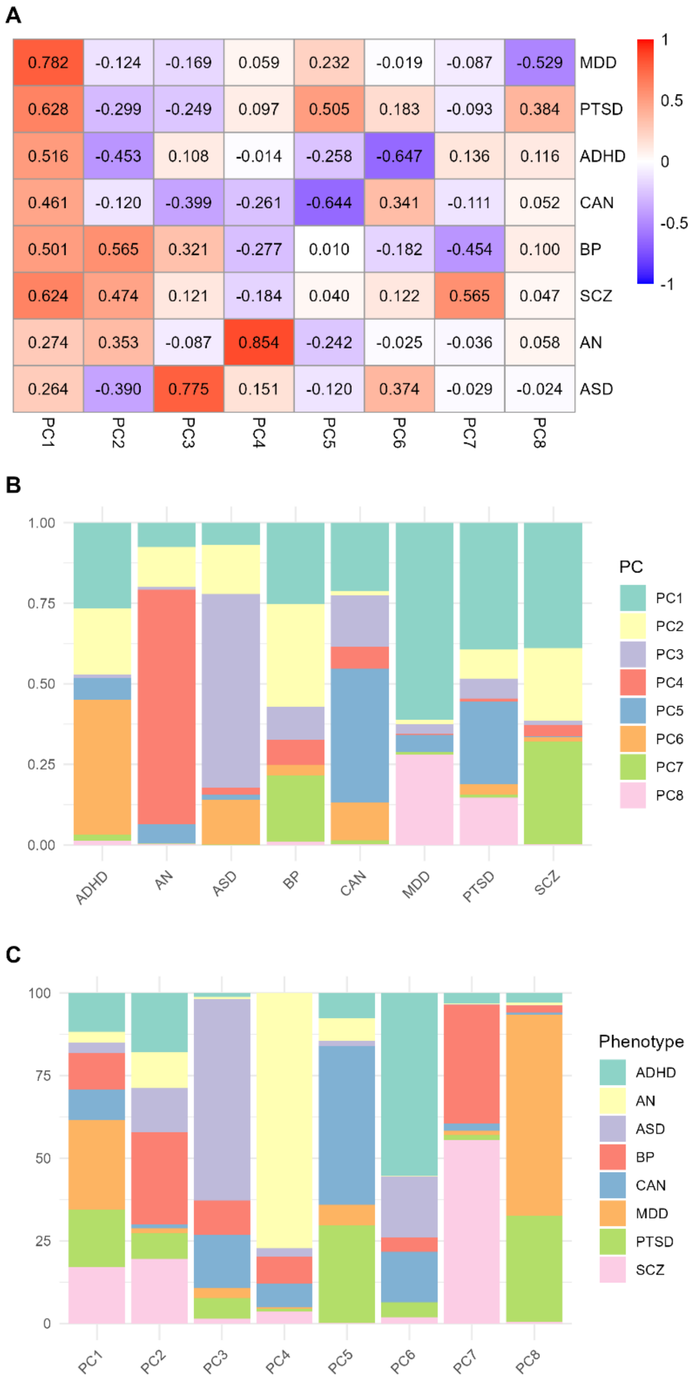

Supplemental figure 15. Characterization of the latent components without FinnGen samples. A) Heatmap of phenotype singular vectors, summarizing the strength of the association between each disorder and each PC. B) Relative contribution of each PC to each disorder. C) Relative contribution of each disorder to each PC.

Signs of PC2 and PC7 were arbitrarily different to the original PCs, although the signs of the eigenvectors have no meaning in SVD.

ADHD, attention deficit hyperactivity disorder; AN, anorexia nervosa; ASD, autism spectrum disorder; BP, bipolar disorder; CAN, cannabis dependence; MDD, major depressive disorder; PC, principal component; PTSD, post-traumatic stress disorder; SCZ, schizophrenia.
